# Promotional Effect of Mn On NH_3_ Synthesis Over Inverse Iron Catalyst

**DOI:** 10.1002/cssc.202502235

**Published:** 2026-02-26

**Authors:** Yuan Jing, Masashi Hattori, Michikazu Hara

**Affiliations:** ^1^ Materials and Structures Laboratory Institute of Science Tokyo Yokohama Japan

**Keywords:** ammonia synthesis, Haber‐Bosch process, heterogeneous catalysis, iron catalyst

## Abstract

The Haber–Bosch process has been established for over a century, and it still remains central to global ammonia production. To this day, industrial NH_3_ synthesis still relies on fuzed iron catalysts developed in the early 20th century, which underscores the ongoing challenge in the development of alternative catalysts with superior activity under mild conditions. Here, we report a Mn‐incorporated iron (FeMn) catalyst prepared via a facile sol–gel method. The resultant FeMn catalyst exhibits an approximately twofold higher NH_3_ formation rate than that for the conventional commercial iron catalyst under mild conditions. Kinetic analysis revealed that Mn incorporation results in ammonia synthesis with a lower apparent activation energy, which suggests enhanced N_2_ activation. Further structural and surface analyses indicate that Mn enrichment at the catalyst surface favors N_2_ activation, which likely facilitates N≡N bond cleavage and contributes to the observed activity enhancement. This work highlights a simple yet effective strategy to boost the performance of Fe‐based catalysts for ammonia synthesis under mild conditions.

## Graphical abstract

We report a novel catalyst with an inverse structure of a supported metal catalyst, consisting of mesoscale iron particles doped with Mn species (FeMn). The FeMn catalyst exhibited about twofold higher NH_3_ formation rate than the conventional commercial iron catalyst under mild conditions. Characterization and kinetic studies revealed that surface Mn_3_N_2_ species play a key role in N_2_ activation, leading to enhanced NH_3_ synthesis over iron.

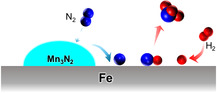

## 1 Introduction

Ammonia produced by the Haber–Bosch (HB) process, typically performed at elevated temperatures (400°C–500°C) and high pressures (10–30 MPa), has not only supported global food production but has also attracted attention as a potential energy carrier [1, 2, 3]. However, reduction of the substantial energy requirement of the HB process remains a century‐old, unresolved challenge.

Significant efforts have been made to address this issue with the aim of enabling efficient ammonia synthesis under low‐temperature and low‐pressure conditions. Ru‐based catalysts have attracted widespread attention since the 1980s due to their superior ability to activate N_2_ at lower temperatures and pressures [4, 5, 6, 7, 8, 9, 10, 11]. However, the practical application of Ru catalysts has been hampered by their scarcity, high cost, and sensitivity to hydrogen poisoning, which together limit their industrial scalability and long‐term stability [12]. As a result, Fe‐based catalysts remain the practical choice for large‐scale ammonia production [13, 14]. Traditional approaches to the enhancement of Fe catalysts have focused on the use of alkali metal promoters—particularly potassium—which increase the electron density on Fe surfaces and facilitate N≡N bond weakening [15, 16, 17]. While effective, these promoters often suffer from volatility, thermal instability, and phase migration under reaction conditions. A wide range of strategies have been explored over the past century to improve Fe‐based catalysts for ammonia synthesis, including supported iron catalysts [18, 19, 20, 21, 22] and Fe alloy catalysts [23, 24, 25]. These efforts have been aimed at enhancement of the catalytic activity under milder conditions or improvement of the stability and tunability. However, despite decades of research, few of these alternatives have demonstrated activity comparable to that of the commercial fuzed iron catalysts used in the HB process. This persistent performance gap underscores the intrinsic challenge in balancing structural accessibility, active site density, and long‐term stability in iron‐based systems.

Against this backdrop, we have focused on mesoscale iron particles with diameters that exceed several tens of nanometers. Ammonia in the HB process is synthesized within a confined reactor volume; therefore, the essential performance metric that governs the energy demand is the ammonia synthesis rate per catalyst volume. In this regard, mesoscale iron particles inherently exhibit greater advantages over supported metal catalysts with lower bulk densities. A prime example that demonstrates this advantage is the commercial fuzed iron catalyst, also known as the doubly promoted iron catalyst [26]. The HB process was industrialized in the early 20th century using this catalyst, which consists of mesoscale iron particles dissolved with various metal oxides such as K_2_O. This classical iron catalyst remains in use at the forefront of current HB plants due to its high synthesis rate per catalyst volume [15].

Although for over a century, no catalyst had surpassed the ammonia synthesis performance of the doubly promoted iron catalyst via enhancement of mesoscale iron particles, recent reports indicate that surface modification of mesoscale iron particles with aluminum hydride can more than double the synthesis rate per catalyst volume compared to the doubly promoted iron catalyst [14]. In this catalyst, aluminum hydride—unexpectedly formed from aluminum oxide species—functions as a strong electron donor that potentially increases the number of active sites on the iron surface. These findings cannot be predicted based on the theoretical framework established through the study of conventional supported metal catalysts, which highlights that surface modification of mesoscale iron particles represents an unexplored frontier in catalysis research. Whether for mesoscale iron particles or supported metal catalysts, the primary strategy toward the enhancement of ammonia synthesis catalysts in the HB process has been the combination of electron‐donating materials with transition metals to increase the electron‐donating capability of the metal center. However, other approaches can also contribute to performance enhancement, such as an increase in the adsorption probability of N_2_ molecules, suppressing hydrogen poisoning by preventing excessive H atom adsorption, and promoting the desorption of ammonia. These functionalities may be more easily implemented in mesoscale iron particles, as modification of the surface of large iron particles is generally easier than modification of the surfaces of metal nanoparticles in supported catalysts.

Here, we report a novel catalyst with a simple inverse structure of a supported metal catalyst that consists of mesoscale Fe particles doped with Mn (FeMn). The FeMn catalyst exhibits significantly enhanced catalytic ammonia synthesis activity at 400°C and atmospheric pressure—conditions far milder than those used in conventional industrial practices.

## 2 Results and Discussion

### 2.1 Structural and Morphological Characterization

**FIGURE 1:**
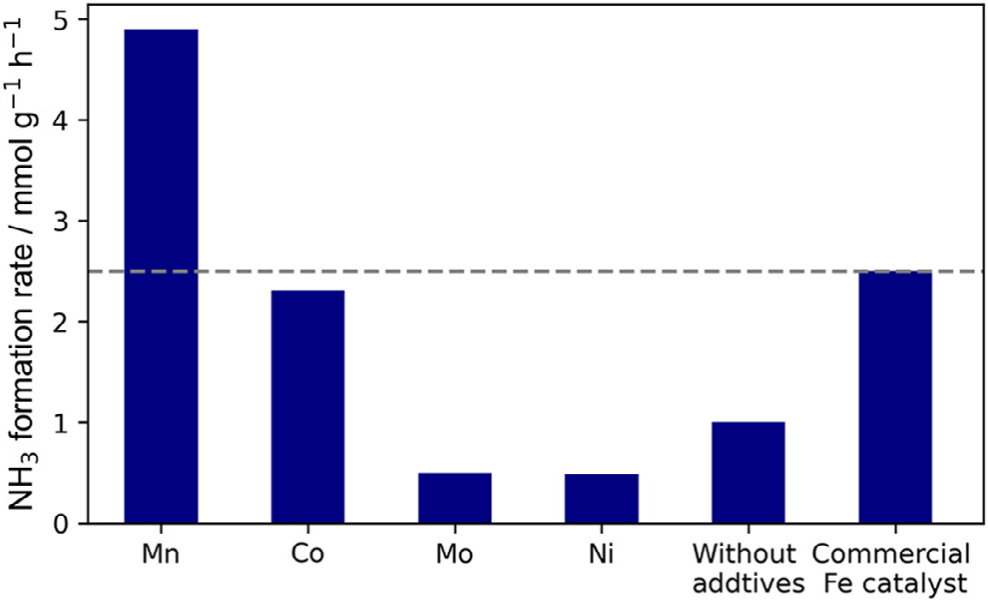
Effect of transition metal on iron catalysts for NH_3_ synthesis at 400°C under atmospheric pressure. Conditions: N_2_/H_2_ = 1:3, total flow rate: 60 mL min^−1^, catalyst weight: 0.1 g.

A series of transition‐metal‐introduced iron catalysts (denoted as FeMO_*x*_, M = Mn, Co, Mo, and Ni) were prepared via a sol–gel method using Fe(NO_3_)_3_·9H_2_O and the corresponding transition metal nitrate as precursors (see details in Experimental Section) [27]. For comparison, pristine *α*‐Fe_2_O_3_ was prepared using the same method. The as‐prepared FeMO_*x*_ catalysts were subsequently reduced under N_2_/H_2_ (1:3) with a total flow rate of 60 mL min^−1^ at 400°C under ambient pressure, which yielded metallic iron catalysts containing incorporated transition metal species. The catalytic performance of the FeMO_*x*_ catalysts in ammonia synthesis was then evaluated at 400°C, as shown in Figure 1. Among these catalyst test additives, Mn exhibited the most significant promotional effect with an NH_3_ formation rate of 4.9 mmol g^−1^ h^−1^, which is almost 5 times that of the Fe‐only catalyst (≈1.0 mmol g^−1^ h^−1^), and notably higher than that of the commercial fuzed iron catalyst (≈2.5 mmol g^−1^ h^−1^). These results highlight the significant promotional effect of Mn on NH_3_ synthesis over the Fe‐based catalyst under mild conditions. Given the outstanding promotional effect, Mn (Fe:Mn = 100:7.5) was then selected for in‐depth investigation in the following study.

**FIGURE 2:**
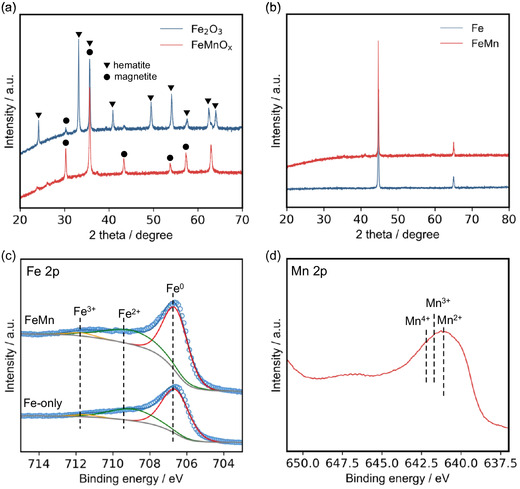
Morphological and structural characterization. XRD patterns of the Fe and FeMn catalysts (a) before and (b) after NH_3_ synthesis reactions. (c) Fe 2*p* and (d) Mn 2*p* XPS spectra for the Fe and FeMn catalysts after reaction.

**FIGURE 3:**
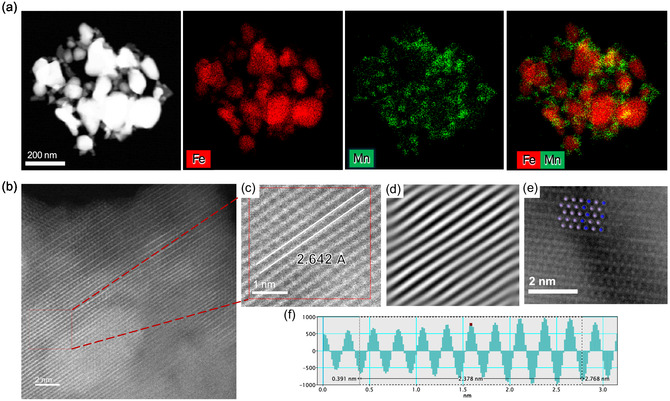
(a) HAADF–STEM image and EDX mapping of FeMn catalyst. (b–f) HAADF–STEM image and corresponding IFFT pattern and IFFT profile.

Figure 2a presents XRD patterns for the oxide precursors, Fe_2_O_3_ and FeMnO_*x*_. The Fe_2_O_3_ sample has diffraction peaks that correspond to *α*‐Fe_2_O_3_, along with weak reflections assignable to Fe_3_O_4_ [28, 29, 30]. The presence of Fe_3_O_4_ may result from the partial reduction of Fe^3+^ during calcination, which is likely induced by the citric acid used in the sol–gel synthesis. In contrast, the FeMnO_*x*_ sample predominantly exhibits diffraction peaks that correspond to Fe_3_O_4_ with no observable peaks for manganese oxides, which indicates that Mn species are homogeneously incorporated into the Fe_3_O_4_ lattice, most likely via substitution at Fe^2+^ sites. Upon activation of the oxide precursors in the mixture of the N_2_/H_2_ (1:3) atmosphere at 400°C (denoted as Fe‐only and FeMn), two sharp peaks appeared at 44.70° and 65.05°, which are assigned to the diffraction peaks for *α*‐Fe. No residual peaks for iron oxide were observed, which indicates complete reduction of the precursors under the activation conditions [13, 29, 31]. The Fe crystallite sizes were calculated using the Scherrer equation and are shown in Table 1. Both the Fe‐only and Mn‐promoted samples exhibit the same crystal phase and comparable crystallite sizes (≈70 nm) after reduction, which indicates that Mn incorporation does not significantly alter the bulk metallic structure of Fe. Figure 2c shows Fe 2*p* X‐ray photoelectron spectroscopy (XPS) spectra of the Fe‐only and FeMn catalysts after reaction. Both samples were transferred into the XPS chamber under an inert atmosphere (glovebox) to prevent exposure to air. The Fe 2*p* XPS spectra of both catalysts are dominated by peaks that correspond to metallic Fe (Fe^0^) [14, 32], which indicates that the metallic phase was retained under the NH_3_ synthesis reaction conditions. The Mn 2*p*_3/2_ peak centered at ≈641 eV indicates that the Mn species in FeMn is predominantly in the +2 oxidation state [33]. The atomic ratio for the surface exposed Fe to Mn was determined to be 1.7 by XPS measurements. High‐angle annular dark‐field scanning transmission electron microscopy (HAADF–STEM) combined with energy dispersive X‐ray spectroscopy (EDX) mapping (Figure 3a) revealed that Fe forms large aggregated particles, which is consistent with the XRD results. While HAADF–STEM observations (Figure 3a) revealed the presence of aggregated species for Mn, the absence of diffraction peaks in the XRD patterns indicates that these segregated Mn species do not form a periodic crystalline structure. High‐resolution STEM imaging (Figure 3b) also revealed well‐defined lattice fringes, and the corresponding fast Fourier transform (FFT) image (Figure 3c), along with the intensity profile analysis (Figure 3f), indicated a lattice spacing of 2.642 Å. This value is close to that for the (110) planes of Mn_3_N_2_. Furthermore, atomic‐resolution imaging (Figure 3e) showed a projected atomic arrangement consistent with the expected crystallographic orientation of Mn_3_N_2_ along the (110) zone axis. While further structural confirmation would be required, these observations may suggest the presence of Mn nitride‐like domains in the FeMn catalyst.

**TABLE 1:** Structural and adsorption characteristics of Fe‐only, FeMn, and commercial Fe catalysts.

Catalyst	*E*_a_, kJ mol^−1^	*S*_BET_, m^2^ g^−1^	Crystallite size, nm^a^	CO uptake, cm^3^ g^−1^	N_2_ uptake, cm^3^ g^−^^1^
FeMn	35	6.7	73.6	0.06	0.53
Fe‐only	50	5.6	71.9	0.17	0.06

a obtained using the Scherrer equation.

### 2.2 Catalytic Performance Evaluation

The NH_3_ formation rate over the FeMn catalysts prepared via the sol–gel method exhibited a clear dependence on the pH of the precursor solution (Figure S1). The highest catalytic activity was achieved at pH = 6, whereas a further increase of the pH to 8 resulted in a significant decrease in activity. This decline may be attributed to the formation of metal hydroxide precipitates under highly alkaline conditions (pH > 7), which could disrupt the homogeneity of the sol and interfere with the development of a uniform oxide gel network during gelation and drying. The influence of the calcination temperature on the NH_3_ formation rate over the FeMnO_*x*_ catalysts is presented in Figure S2. The catalytic activity peaked at a calcination temperature of 450°C, beyond which a gradual decline was observed. This reduction in activity is likely attributable to the sintering of FeMnO_*x*_ at elevated temperatures, which leads to a loss of the active surface area. Concurrently, the Mn content played a crucial role in modulating the catalytic performance (Figure S3). A systematic variation of the Fe/Mn ratio revealed a pronounced dependence of the NH_3_ formation rate on the Mn loading, with the highest activity achieved at an Fe:Mn ratio of 100:7.5. Based on these findings, the optimal synthesis conditions were identified as a precursor pH of 6, a calcination temperature of 450°C, and a Fe/Mn molar ratio of 100:7.5. The catalyst prepared under these conditions exhibited the highest NH_3_ formation rate among all the tested samples, and was therefore selected as the representative FeMn catalyst for further mechanistic investigation into the promotional role of Mn.

**FIGURE 4:**
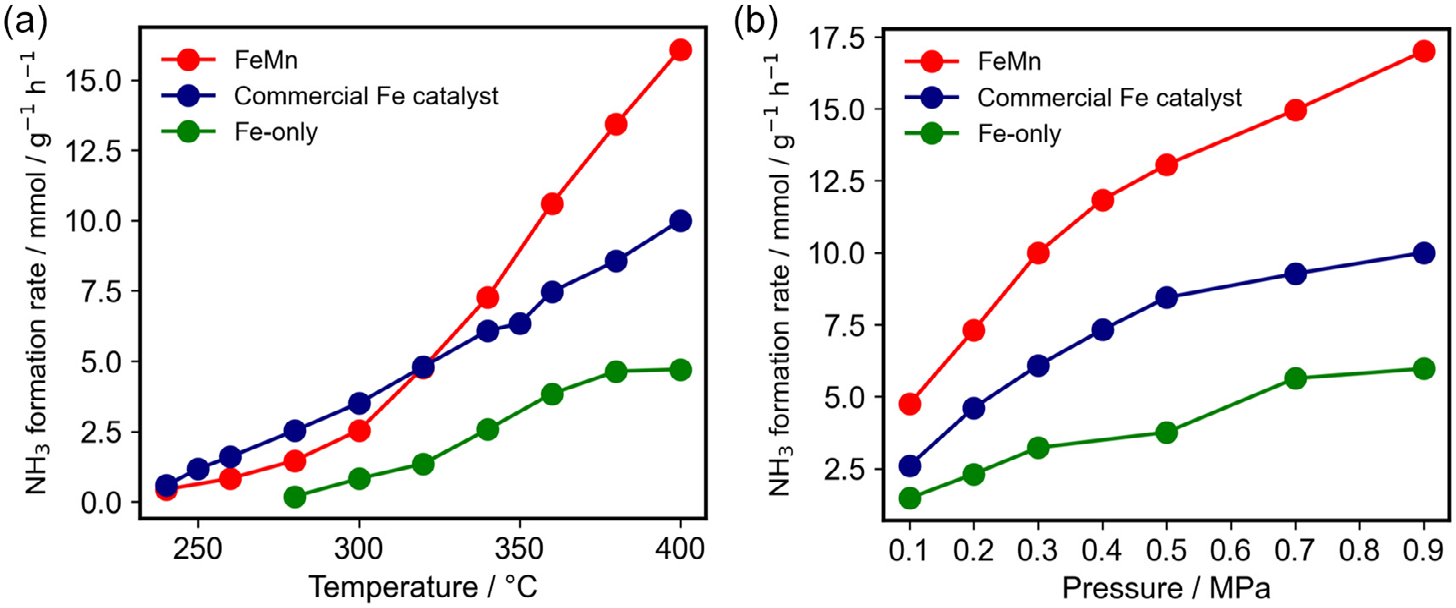
Catalytic NH_3_ synthesis performance over Fe‐only, FeMn, and commercial Fe catalysts. (a) Dependence of NH_3_ formation rate on temperature at 0.9 MPa. (b) Dependence of ammonia formation rate on reaction pressure at 400°C.

The catalytic performance of the FeMn catalyst for NH_3_ synthesis was further evaluated under different reaction conditions and compared with that of the Fe‐only control catalyst and a commercial promoted iron catalyst that consisted of Fe, K_2_O, Al_2_O_3_, and CaO [13, 14]. Figure 4a shows that under a reaction pressure of 0.9 MPa, the FeMn catalyst consistently outperformed the Fe‐only sample across the entire tested temperature range, which suggests the promotional effect of Mn on NH_3_ formation. The catalytic performance also surpassed that of the commercial Fe catalyst, particularly at temperatures above 320°C. Figure 4b illustrates the dependence of the NH_3_ formation rate on the reaction pressure at 400°C. The FeMn catalyst exhibited a higher NH_3_ formation rate to that for the Fe‐only and commercial Fe catalysts across the entire pressure range. In contrast to the Fe‐only sample, which showed limited enhancement at elevated pressures, the FeMn catalyst maintained a steady increase in performance, which suggests improved responsiveness to the reaction pressure. The rates of ammonia synthesis per catalyst volume of the FeMn, commercial Fe, and Fe‐only catalysts at 400°C and 0.9 MPa were estimated to be 28.4, 16.7, and 9.9 mmol h^−1^ mL^−1^, respectively [14]; the FeMn catalyst has much higher ammonia synthesis activity per catalyst volume than the commercial Fe catalyst. As a result, replacement of the commercial Fe catalyst in the HB process reactor with the FeMn catalyst means that the amount of ammonia produced by the reactor increases by 1.7 times, while the energy required for ammonia synthesis decreases by ≈40%. Such a catalyst that surpasses the commercial Fe catalyst in ammonia synthesis activity per catalyst volume has not been reported, except for a few rare exceptions. One such exception is mesoscale Fe particles loaded with aluminum hydride [14]. A notable feature of Figure 4b is that the rate of ammonia synthesis over the commercial Fe catalyst at 0.9 MPa can be achieved using only the FeMn catalyst at 0.3 MPa. The energy consumption of the HB process itself is defined as the energy required for nitrogen production, compression, and ammonia cooling/separation, minus the heat generated during ammonia synthesis. Compression accounts for a significant portion of the energy consumption in the HB process. Moreover, when the amount of ammonia produced is the same, the energy required for nitrogen production, compression, and the heat generated during synthesis remains constant. Therefore, it is clear that the energy consumed for compression determines the overall energy consumption of the process. For this reason, the FeMn catalyst, which can achieve an ammonia synthesis rate comparable to that for the commercial Fe catalyst at one‐third of the pressure, is expected to significantly reduce the energy required for ammonia production. The FeMn catalyst produced ammonia without a decrease in activity even after reaction for over 100 h (Figure S4). The stability of the FeMn catalyst was further evaluated by a temperature cycling test, which shows that the ammonia formation rate is well reproduced during heating and cooling processes, indicating good catalytic stability (Figure S5).

### 2.3 Mechanistic Investigations

**FIGURE 5:**
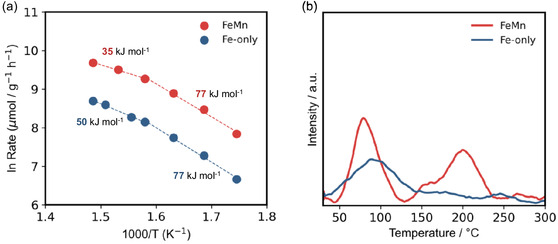
(a) Arrhenius plot for Fe and FeMn catalyst in temperature range of 300°C–400°C at 0.9 MPa. (b) Profile of the CO‐TPD measurement.

To obtain mechanistic insight into the role of Mn in NH_3_ synthesis, the apparent activation energy (*E*_a_) was estimated from Arrhenius plots derived from the NH_3_ formation rates. The Arrhenius plots below 360°C shown in Figure 5a indicate that while the FeMn catalyst showed a similar *E*_a_ to the Fe‐only catalyst in the low‐temperature region, the former had a larger natural logarithm term than the latter. On the other hand, the FeMn catalyst showed a lower activation energy than the Fe‐only catalyst at temperatures above 360°C, although the natural logarithm of FeMn exceeded that of the Fe‐only catalyst. This means that FeMn is superior to the Fe‐only catalyst in terms of the frequency factor in the Arrhenius equation and Mn‐induced modifications of Fe result in a smaller *E*_a_ above 360°C.

Further elucidation of the promotional effect of Mn was conducted using a series of characterization techniques to assess the surface exposure and adsorption behavior. N_2_ physisorption measurements revealed that the FeMn catalyst possessed a slightly larger Brunauer–Emmett–Teller (BET) surface area (6.7 m^2^ g^−1^) than the Fe‐only catalyst (5.6 m^2^ g^−1^) (Figure S6 and Table 1). Furthermore, CO was employed as a probe for monitoring changes in the adsorption characteristics of the catalyst surface. CO and N_2_ are isoelectronic molecules that both possess a triple bond and exhibit similar electronic and adsorption behavior on transition metal surfaces. CO is widely used as a surrogate to probe for N_2_ activation properties due to its stronger IR activity and ease of detection. Post‐reaction CO chemisorption measurements showed that the CO uptake on the Fe‐only and FeMn catalysts was 0.17 and 0.06 cm^3^ g^−1^ (Table 1), respectively. The significantly lower CO adsorption capacity observed for the FeMn catalyst suggests that Mn may alter the electronic properties or adsorption sites of Fe, which would weaken the binding strength of strongly adsorbed species such as CO (and by analogy, N_2_). In contrast, N_2_ chemisorption revealed that the FeMn catalyst exhibited significantly higher N_2_ uptake (0.53 cm^3^ g^−1^) compared with the Fe‐only catalyst (0.06 cm^3^ g^−1^) (Table 1). Considering that surface characterization indicated the possible presence of Mn_3_N_2_‐related structures and that the sample was subjected to H_2_ reduction at 400°C, it is plausible that nitrogen defects associated with Mn‐containing phases capture and stabilize additional N_2_ molecules.

XPS analysis further confirmed notable differences in the surface composition. The surface atomic ratios Fe^0^/Fe^2+^/Fe^3+^/Mn^2+^/O (FeMn) and Fe^0^/Fe^2+^/Fe^3+^/O (Fe‐only) were determined to be 17/8/1/15/59 and 19/8/1/72, respectively (Table S1). These results indicate that the number of Fe^0^ atoms on the surface of the FeMn catalyst is equal to or less than that on the surface of the Fe‐only catalyst. Nevertheless, FeMn surpasses the Fe‐only catalyst in the frequency factor in the Arrhenius equation, which implies that Mn incorporation partially covers Fe active sites while new adsorption environments are simultaneously formed.

Taken together, these observations clearly demonstrate that the enhanced NH_3_ synthesis activity of the FeMn catalyst is not attributable to an increase in the number of exposed Fe sites, but rather to modifications in the adsorption properties. CO temperature‐programed desorption (CO‐TPD) measurements were then performed to further examine this interpretation, and the results are shown in Figure 5b. The Fe‐only catalyst exhibits a single major CO desorption peak centered at around 100°C, which corresponds to relatively weak adsorption sites. In contrast, the FeMn catalyst shows two distinct peaks: a low‐temperature peak similar to that of the Fe‐only catalyst, and an additional high‐temperature peak centered around 200°C. The emergence of this high‐temperature desorption signal indicates the formation of stronger CO adsorption sites upon Mn incorporation, which may be responsible for the increase in the frequency factor in the Arrhenius equation for FeMn.

Table 2 summarizes the reaction orders at 400°C for the FeMn and Fe‐only catalysts (Figure S7). The reaction order for H_2_ remained essentially unchanged upon Mn incorporation (1.67 for Fe vs. 1.64 for FeMn), which indicates that the hydrogenation steps in the reaction pathway are not significantly affected. On the other hand, the FeMn catalyst exhibits a smaller reaction order with respect to N_2_ than the Fe‐only catalyst, which indicates that the former is superior to the latter in N_2_ dissociative adsorption. This improvement is evidently related to the Mn species, and one plausible interpretation is that Mn enhances electron donation to adsorbed N_2_ molecules through Fe, thereby facilitating N_2_ cleavage. In the case of the commercial Fe catalyst, the strong electron donation power of K_2_O with a small work function (1.35 eV) [34] facilitates N_2_ cleavage as the rate‐determining step for ammonia synthesis, which results in a small *E*_a_ (30–35 kJ mol^−1^) [14, 35]. HAADF–STEM observations (Figure 3) suggested the formation of Mn_3_N_2_ on FeMn. Although the work function for Mn_3_N_2_ has not been reported, it can be estimated to be not significantly different from that for metallic Fe (≈4.5 eV) by analogy with other transition‐metal nitrides (e.g., TiN, TaN, NbN, and HfN) [36, 37, 38]. Therefore, while Mn incorporation may influence the electronic state of Fe, the presence of Mn_3_N_2_ itself is unlikely to provide the strong electron donation required to account for the small *E*_a_ of FeMn, even though its *E*_a_ is comparable to that for the commercial Fe catalyst and much lower than that for the Fe‐only catalyst (50 kJ mol^−1^). Ertl et al. demonstrated that the true activation energy for N_2_ cleavage, the rate‐determining step in ammonia synthesis over iron catalysts, is only several kilojoules per mole [15, 39, 40]. However, the measured *E*_a_ generally increases up to 30–35 kJ mol^−1^ because the apparent *E*_a_ reflects not only the activation energy for the rate‐determining step, but also contributions from various effects, such as mass transport, surface adsorption capacity for reactants, and product desorption ability. In this context, phenomena other than N_2_ cleavage may contribute to the small *E*_a_ observed for the FeMn catalyst.

**TABLE 2:** Apparent reaction order for N_2_, H_2_, and NH_3_ for Fe‐only and FeMn catalysts obtained at 400°C under reaction pressure of 0.9 MPa.

Sample	N_2_	H_2_	NH_3_
FeMn	0.86	1.64	−1.50
Fe‐only	1.34	1.67	−2.03

One possible explanation for the role of Mn species on the FeMn catalyst is strong N_2_ adsorption. CO‐TPD (Figure 5b) measurements implied that the FeMn catalyst adsorbs CO more strongly than the Fe‐only catalyst, which suggests that FeMn also strongly adsorbs N_2_ molecules. This could be the reason for the small *E*_a_ for the FeMn catalyst. The higher N_2_ uptake for the FeMn catalyst (Table 2) further suggests that these strong adsorption sites are associated with nitrogen vacancies in Mn_3_N_2_. Such vacancies could reasonably contribute to N_2_ activation, given the strong affinity of early 3*d* transition metals for nitrogen species. The activated nitrogen species at Mn‐related sites may subsequently react with hydrogen species adsorbed and activated on Fe, which would facilitate NH_3_ formation. Notably, similar vacancy‐enabled N_2_ activation has been established as an effective approach for NH_3_ synthesis; for example, Ni‐loaded LaN was reported to activate N_2_ at nitrogen‐vacancy sites while the metal component dissociates H_2_, resulting in a dual‐site mechanism [41]. A subsequent study further proposed a general guideline for metal nitride catalysts in which the nitrogen vacancy formation energy governs the catalytic performance, further highlighting the importance of nitrogen vacancies in facilitating N_2_ activation [42]. Specifically, catalyst comprised of Mn nitrides and alkali metal hydrides has been reported to promote NH_3_ synthesis, in which the Mn nitrides serve as the active phase for N_2_ activation, while the hydrides facilitate subsequent hydrogenation [43]. Likewise, Mn nitrides have been employed as supports for Ni and Fe catalysts, and density functional theory (DFT) studies have revealed the key role of the nitrogen vacancies in Mn_3_N_2_ in N_2_ activation, with transition‐metal components mainly responsible for H_2_ activation [44]. In light of these previous reports, the present results suggest that a Mn_3_N_2_‐related phase in the FeMn catalyst is likely involved in N_2_ activation through nitrogen vacancies, with Fe sites serving as the primary sites for hydrogen activation. With respect to other possible mechanisms, such as Mn‐induced phase transformation or surface reconstruction, no experimental evidence supporting these effects is observed in the present system. XRD analysis shows that Fe remains in the *α*‐Fe phase after Mn introduction, identical to that of the Mn‐free Fe catalyst, without any detectable peak shift. In addition, XPS measurements indicate that Mn is predominantly present Mn^2+^, while the Fe 2*p* spectra of the FeMn catalyst remain dominated by metallic Fe features, comparable to those of the Fe‐only catalyst. These results suggest that the addition of Mn does not induce detectable bulk phase transformation or pronounced surface reconstruction within the resolution of the applied techniques. Accordingly, based on the available experimental results, nitrogen vacancies associated with Mn_3_N_2_ are considered to provide one possible explanation for the promotional effect induced by Mn addition. Notably, a reduced apparent activation energy upon Mn addition is observed only in the high‐temperature regime, while no significant difference is detected at lower temperatures compared with the Fe‐only catalyst. This result supports the proposed mechanism, in which the Mn‐related contribution becomes operative only when sufficient thermal energy is available to overcome the kinetic barriers associated with the migration of adsorbed species.

## 3 Conclusions

In summary, we have prepared a series of transition‐metal‐incorporated iron catalysts via a simple sol–gel method. Mn exhibited an outstanding promotional effect on NH_3_ synthesis for the iron catalyst. The as‐prepared FeMn catalyst exhibited high efficiency for NH_3_ synthesis under mild conditions and catalytic activity that was twice as high as that for a fuzed iron‐based catalyst. The results of characterization and kinetic studies indicate that the incorporation of Mn induces the formation of stronger adsorption sites for N_2_, which are highly efficient for the activation of N_2_ and lead to the significant enhancement of NH_3_ synthesis over the iron catalyst. Taken together, the present results provide direct experimental evidence that transition‐metal doping—without the use of alkali promoters—can effectively modulate the surface reactivity of Fe, and thus offer a mechanistically distinct route to catalyst design. This work not only deepens our understanding of the Fe–Mn interaction in N_2_ activation but also suggests a practical and scalable pathway toward high‐performance ammonia synthesis under decentralized, energy‐efficient conditions. The insights gained from the promotional effect of Mn species may further inspire the design and development of heterogeneous catalysts for other chemical processes.

## 4 Experimental Section

### 4.1 Preparation of Materials and Catalysts

A series of transition‐metal‐introduced iron catalysts was prepared via the citrate sol–gel method using iron nitrate nonahydrate (Fe(NO_3_)_3_ **·**9H_2_O) and transition metal nitrates as the metal precursors [27]. Typically, 0.01 mol of Fe(NO_3_)_3_ **·**9H_2_O and the corresponding amount of transition metal nitrate were dissolved in 50 mL of de‐ionized water. After stirring for 30 min, citric acid was then added to the mixture with further stirring for 30 min, where the molar ratio of citric acid to total metal precursors was 1.2:1. Ammonia solution (NH_4_OH, pH = 12) was then added dropwise into the solution until the pH value reached 6.0. The solution was subsequently heated at 80°C for 2 h followed by drying at 120°C overnight to generate a dry gel. The obtained gel was then calcined at 450°C with a ramp rate of 3°C min^−1^. The Fe_2_O_3_ catalyst was also prepared using the same sol–gel method without addition of the Mn precursor.

### 4.2 Catalyst Characterization

XRD measurements were conducted using Cu Ka radiation (Miniflex600C, Rigaku). After ammonia synthesis, the catalysts were collected in an Ar‐filled glove box and then set in a sealed XRD sample holder. The sealed catalysts were subsequently subjected to XRD measurements without exposure to air. Nitrogen adsorption measurements were obtained using a BELSORP‐mini apparatus (MicrotracBEL) at −196°C to obtain the BET surface areas. HAADF–STEM and EDX measurements were performed using a JEOL JEM‐ARM 200F microscope. CO‐TPD measurements were conducted using a BELCAT instrument (MicrotracBEL). XPS measurements were performed using a Shimadzu ESCA‐3200 spectrometer (Mg K*α*, 8 kV, 30 mA). The binding energy was calibrated by setting the C 1*s* signal at 248.8 eV.

### 4.3 Catalytic Activity Evaluation

Evaluation of the catalytic activity was conducted in a stainless‐steel fix‐bed flow reactor at 400°C under a N_2_/H_2_ gas mixture (N_2_:H_2_ = 1:3) with a total flow rate of 60 mL min^−1^ under atmospheric pressure, where the amount of the Fe_2_O_3_ and FeMnO_*x*_ catalysts was ≈0.14 g (iron catalyst ≈ 0.1 g). Activation was conducted at 400°C under the above conditions with a ramp rate of 3°C min^−1^. After the steady state was reached, the ammonia formation rate was evaluated under various reaction conditions.

During evaluation of the catalytic ammonia synthesis rate, the ammonia in the outlet of the reactor was trapped in 5 mM H_2_SO_4_ aqueous solution, and the concentration of the generated NH_4_^+^ was estimated using ion chromatography (LC‐2000 plus, Jasco) with a thermal conductivity detector. The rate of ammonia synthesis was measured 3 times to ensure that the obtained ammonia synthesis rate had an error of less than 5%.

## Supporting Information

Additional supporting information can be found online in the Supporting Information section. **Supporting Fig. S1:** Effect of the pH value of the sol in sol–gel method on the NH_3_ formation rate of FeMnO*_x_* catalyst. Conditions: 0.1 MPa, 400°C, total flow rate: 60 mL min^−1^ (N_2_:H_2_ = 1:3), catalyst weight: 0.1 g. **Supporting Fig. S2:** Dependence of the NH_3_ formation rate on the calcination temperature. Conditions: 0.1 MPa, 400°C, total flow rate: 60 mL min^−1^ (N_2_:H_2_ = 1:3), catalyst weight: 0.1 g. **Supporting Fig. S3** **:** Dependence of the NH_3_ formation rate on the amount of Mn in FeMnO*_x_* catalyst. **Supporting Fig. S4** **:** Results of the catalytic stability test of FeMn catalyst. Conditions: 0.1 MPa, 400°C, total flow rate: 60 mL min^−1^ (N_2_:H_2_ = 1:3), catalyst weight: 0.1 g. **Supporting Fig. S5** **:** Results of the cycling test of FeMn catalyst. Conditions: 0.1 MPa, total flow rate: 60 mL min^−1^ (N_2_:H_2_ = 1:3), catalyst weight: 0.1 g. **Supporting Fig. S6** **:** Isotherm of N_2_ adsorption for FeMnO*_x_* after reaction. **Supporting Fig. S7** **:** Dependence of the NH_3_ formation rate on the particle pressure of (a) H_2_ and (b) N_2_. **Supporting Table S1:** Surface composition of Fe and FeMn obtained by XPS spectra.

## Funding

This study was supported by Japan Society for the Promotion of Science (23H00245, 24K23012, 25K17886).

## Conflicts of Interest

The authors declare no conflicts of interest.

## Supporting information

Supplementary Material

## Data Availability

The data that support the findings of this study are available in the supplementary material of this article.
