# Supplementary material for: Promotional Effect of Mn On NH3 Synthesis Over Inverse Iron Catalyst
Source: ChemSusChem. 2026 Feb 26;19(5):e202502235. doi: 10.1002/cssc.202502235 (PMC12936808; doi:10.1002/cssc.202502235)
Supplement: Supplementary file 1 — Supplementary Material [file CSSC-19-e202502235-s001.pdf]

Supporting information

Promotional effect of Mn on NH_3 synthesis over inverse iron catalyst

Yuan Jing, Masashi Hattori, Michikazu Hara*

Materials and Structures Laboratory, Institute of Science Tokyo, 4259 Nagatsuta, Midori-ku, Yokohama 226-8503, Japan

Email: hara.m.3df4@m.isct.ac.jp

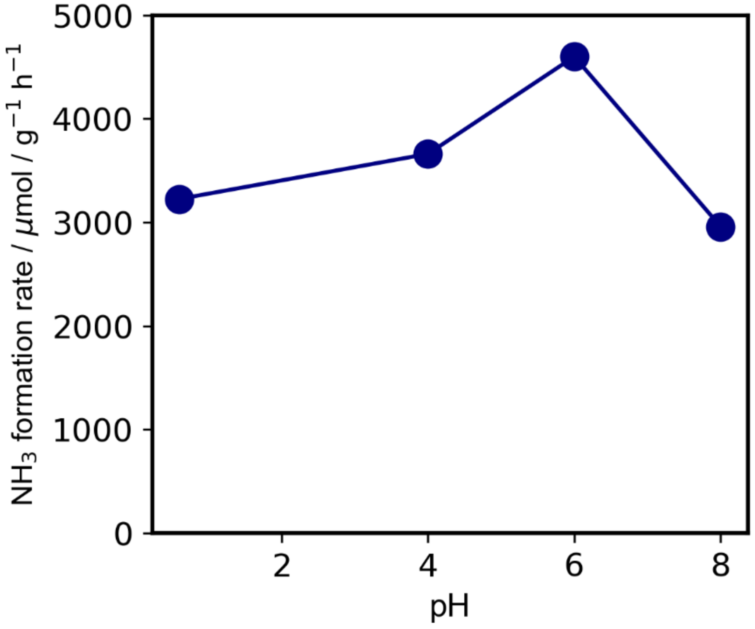

Figure S1. Effect of the pH value of the sol in sol-gel method on the NH_3 formation rate of $FeMnO_x$ catalyst. Conditions: 0.1 MPa, 400 °C, total flow rate: 60 ml min^{-1} (N_2:H_2=1:3), catalyst weight: 0.1g.

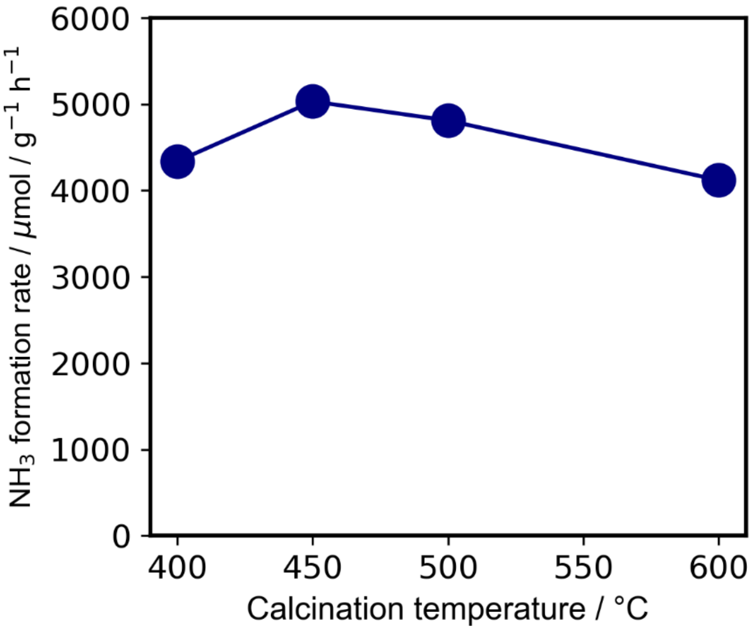

Figure S2. Dependence of the NH_3 formation rate on the calcination temperature. Conditions: 0.1 MPa, 400 °C, total flow rate: 60 ml min^{-1} (N_2:H_2=1:3), catalyst weight: 0.1g.

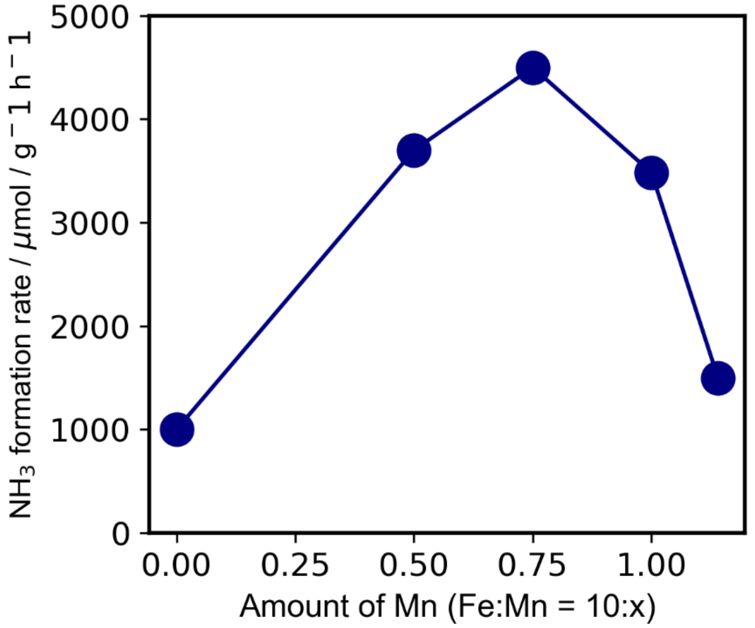

Figure S3. Dependence of the NH_3 formation rate on the amount of Mn in $FeMnO_x$ catalyst.

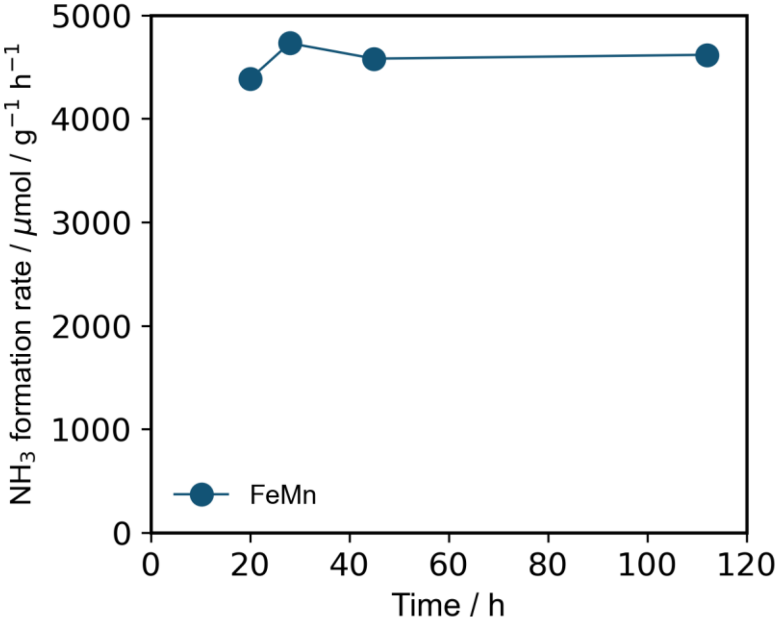

Figure S4. Results of the catalytic stability test of FeMn catalyst. Conditions: 0.1 MPa, 400 °C, total flow rate: 60 ml min^{-1} (N_2:H_2=1:3), catalyst weight: 0.1g.

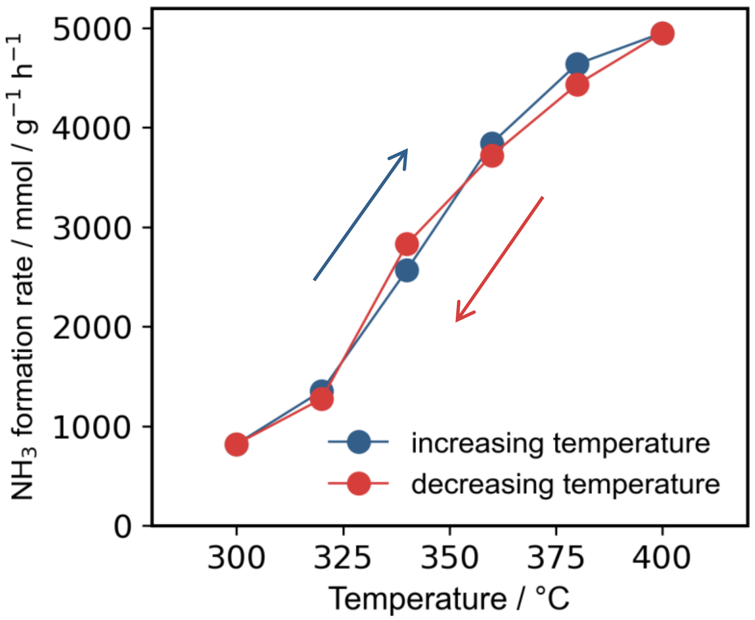

Figure S5. Results of the cycling test of FeMn catalyst. Conditions: 0.1 MPa, total flow rate: 60 ml min^{-1} (N_2:H_2=1:3), catalyst weight: 0.1g.

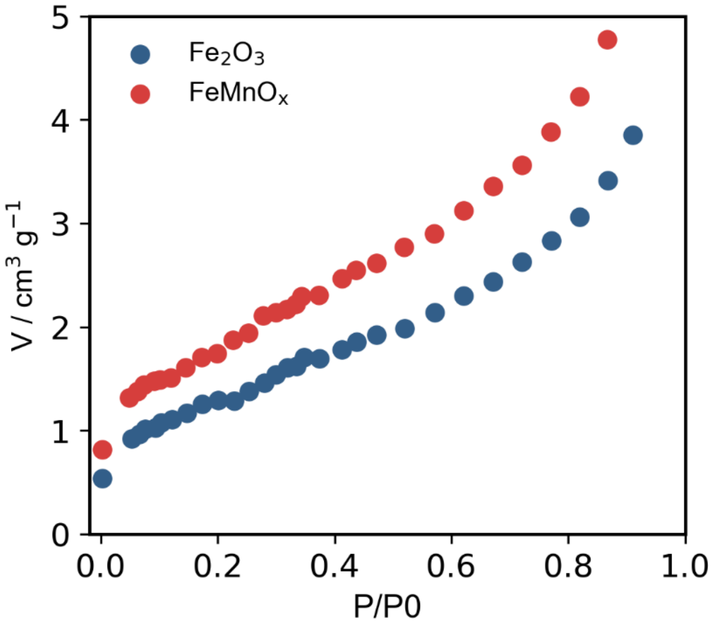

Figure S6. Isotherm of N_2 adsorption for $FeMnO_x$ after reaction.

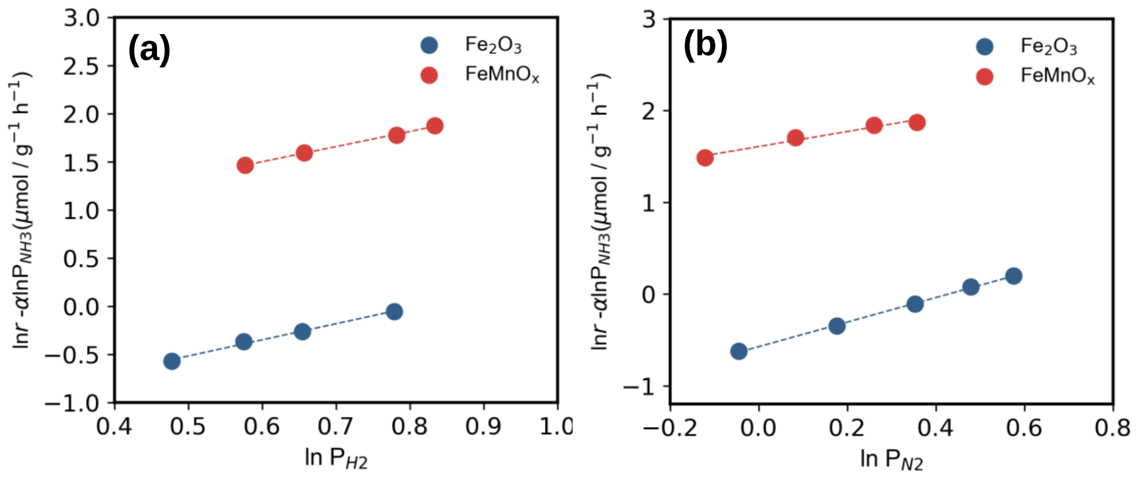

Figure S7. Dependence of the NH_3 formation rate on the particle pressure of (a) H_2 and (b) N_2.

Table S1. Surface composition of Fe and FeMn obtained by XPS spectra.

Catalyst	Fraction / %				
	Fe^0	Fe^{2+}	Fe^{3+}	Mn^{2+}	O
Fe	19	8	1	-	72
FeMn	17	8	1	15	59
